# Ni justo ni legítimo: The role of social status and neoliberal context on perceived social justice in Latin America and its political consequences

**DOI:** 10.1111/bjso.12894

**Published:** 2025-04-28

**Authors:** Dante Solano‐Silva, César Guadalupe, Eileen Sam‐Castañeda

**Affiliations:** ^1^ University of Leeds Leeds UK; ^2^ Universidad del Pacífico Lima Peru

**Keywords:** Latin America, neoliberalism, perceived social justice, self‐interest, social status

## Abstract

We study the role of social status and neoliberal contexts on perceptions of social justice and their consequences for political behaviour in Latin America. While most literature measures these perceptions through personal assessments of income distribution fairness, we resort to a wider understanding including perceptions of fairness on access to fundamental rights. Using data from the Latinobarómetro 2020 survey (N ≈ 20,204), we find that perceived social justice involves assessments of income inequality and access to education, health and justice. Based on self‐interest theory, we expect higher‐status individuals to perceive the world as more just, as this perception aligns with their interests. We also argue that neoliberal contexts moderate this relationship by narrowing justice perception gaps across status groups, as these contexts advance ideologies emphasizing individual responsibility and meritocracy. Regression models suggest that social status, measured through three indicators (social class, socioeconomic status, and subjective income) is positively related to perceived social justice. Interaction models suggest that, in more neoliberal societies, differences in perceived social justice among social status groups tend to narrow. Finally, we find that perceived social justice is associated with satisfaction with life, democracy and economic system, and reduced intention to participate in protests.

This research aims to study the antecedents and consequences of perceptions of social justice in Latin America, taking a broader conceptualization of perceived social justice that includes access to fundamental rights. Social justice has primarily been a philosophical discussion about how resources, opportunities and rights are fairly distributed in societies (Jost & Kay, 2010; Tyler, 2015). Social and political psychologists have focused on individuals' perceptions of social fairness, as these assessments have been related to relevant political outcomes (e.g. García‐Sánchez et al., 2024). The main literature has concentrated on people's evaluations of distributive justice in their societies—particularly income distribution perceptions (Caricati, 2017; Castillo et al., 2022; García‐Sánchez et al., 2022). This approach presents significant limitations for understanding how people process information about fairness in their environment, especially in developing contexts where inequities extend beyond income (Soler‐Martínez et al., 2023).

Concerning antecedents, we argue that social status is positively related to social justice perceptions and further hypothesize that this relationship is moderated by the degree to which neoliberal policies have been adopted within national contexts. Following self‐interest theory (Miller, 1999), people of higher social status tend to perceive societies as fairer, as this allows them to have a more coherent view of the society from which they get their benefits (Rodriguez‐Bailon et al., 2017). At a societal level, the level of income inequality has been indicated as related to unfair perceptions of distributive justice (Reyes & Gasparini, 2022). However, considering recent advances in the psychological consequences of neoliberalism, it is relevant to study whether this development policy is a moderator of justice perceptions. Neoliberal policies (e.g. deregulation, promotion of private investments, etc.) promote ideas of individual responsibility and meritocracy, which may affect perceptions of equality and social justice (Adams et al., 2019; Bettache et al., 2020; England & Ward, 2016). We hypothesize that the extent to which societies have embraced neoliberal policies would moderate the disparity in justice perceptions between different social status groups. However, this possible link between perceptions of social justice and countries' adoption of neoliberal policies has been little explored in the literature.

Regarding political consequences, this study suggests that perceptions of social justice are linked to attitudes that legitimize the system and to the pursuit of social change (Kesberg et al., 2024). In this vein, previous research suggests that fairness perceptions are related to social and political trust (García‐Sánchez et al., 2024), life satisfaction (Jia et al., 2020), and support and participation in protests (Reyes & Gasparini, 2022; Soler‐Martínez et al., 2023). We extend this evidence by testing the relationship between perceived social justice and satisfaction with democracy and the economic system, and the attitudes toward politicians.

We are interested in testing these objectives in Latin America, a region that offers an interesting scenario for this research since it is characterized by high levels of economic and social disparities (ECLAC, 2023), recent events of political turmoil and a long‐standing debate around neoliberalism (Murillo, 2021; Ruckert et al., 2017). The prevalence of high inequalities among these societies has led many researchers to look at the association between this type of structural inequality and perceptions of income justice in this region, finding a positive association (e.g., García‐Sánchez et al., 2024; Reyes & Gasparini, 2022; Zmerli & Castillo, 2015). This region has also been marked by the debate around the adoption of neoliberalism as the main economic development policy. Since its introduction during the dictatorship of Pinochet in Latin America, neoliberal policies expanded and maintained across almost the entire region during the following decades (Ruckert et al., 2017).

To meet our objectives, we used data from the Latinobarómetro 2020. This survey collects different perceptions and attitudes on social, economic, and political issues in representative samples from 18 countries in the Latin American region. Variables associated with perceptions of social justice, social status, and other political behaviours were produced by this survey. To measure variables at the societal level, we used data from the United Nations (Human Development Index), Economic Commission for Latin America (Gini Index), and the Heritage Foundation (Neoliberalism—Economic Freedom Index). Using exploratory, confirmatory, and multigroup factor analyses, we develop a measure of perceived social justice for Latin American societies. Then, we develop a series of multilevel models to test the relationship of antecedents and consequences of perceptions of social justice.

This research contributes to the current literature and Latin American politics in different aspects. First, it broadens the way perceptions of social justice are measured by including aspects that go beyond income distribution. In a context of long‐standing inequities such as Latin America, this becomes relevant. Second, this research expands on the antecedents usually studied in the literature and reviews the role of neoliberalism in these perceptions, something that has not necessarily been previously reviewed. Third, this research also broadens the field of political psychology research, where more evidence is needed from developing contexts (Rivera Pichardo et al., 2022). Finally, this research can contribute to understanding the sociopolitical processes that have taken place in Latin America in recent years, which have been marked by political turbulence and protests (Murillo, 2021). This study, therefore, will contribute from social and political psychology to look at how subjectivities also played an important role in the political instability of this region.

The article is structured as follows. The next section discusses the concept of social justice perceptions. This is followed by the discussion on the antecedents of these perceptions, focusing on social status and neoliberalism, to later comment on the possible consequences of these perceptions. The next section presents the objectives of the study within the framework of the Latin American context. Subsequently, the methods used to analyse the antecedents and consequences of perceptions of justice are detailed, followed by the results of these statistical analyses. Finally, the last section provides the study's conclusions.

## Perceptions of social justice

The concept of social justice encompasses the debate about what makes a society qualify as socially just. The main authors suggest that this concept is comprised of at least two types of justice (Jost & Kay, 2010; Tyler, 2015): distributive and procedural justice. The first type refers to how resources and benefits are fairly distributed in a society, while the second type involves how procedures, norms, and rules preserve people's rights and freedoms (Jost & Kay, 2010; Tyler, 2015). From the perspective of social psychology, these concepts have been studied through the subjective evaluations that people make about these types of justice (Colquitt & Rodell, 2015; Jia et al., 2020). Thus, perceptions of social justice refer to people's evaluations of how resources, opportunities and rights are fairly given in a society (Jia et al., 2020).

Perceptions of justice are formed through the cognitive capacity of individuals to compare their views of actual rewards versus their ideas of just rewards (Jasso, 2015) or by their views about the processes of distribution of actual rewards (Lind & Tyler, 1988) on a particular event. Individuals evaluate these aspects of justice based on moral principles such as equity, merit or need (Colquitt & Rodell, 2015; Jasso, 2015). The formation of these perceptions is also influenced by affective elements of individuals (Van Den Bos, 2003), as well as by their experiences from their socio‐cultural environment (Janmaat, 2013). These conceptions of social justice evaluations mainly rely on socio‐psychological theories such as equity theory (Adams, 1965), social comparison theory (Festinger, 1954), or relative deprivation theory (Runciman, 1972). These theories emphasize that justice perceptions are inherently comparative and contextual rather than absolute judgements. Furthermore, individuals are motivated to generate these justice evaluations to justify social inequalities and the status quo, which allows them to give coherence and certainty to their social world (Jost, 2019; Lerner, 1980). Once people have formed their perceptions of justice, they serve as heuristics for social decision‐making (Lind, 2001).

Scholars in social sciences have usually focused on subjective evaluations of distributive justice like fairness perceptions of income inequalities and their consequences (e.g. Castillo et al., 2022; García‐Sánchez et al., 2024; Reyes & Gasparini, 2022). However, this type of measurement has significant limitations since income disparities do not capture several dimensions of social inequality (Soler‐Martínez et al., 2023). Social justice research will be enriched with studies that incorporate evaluations of justice on procedural aspects such as access to basic rights (Colquitt & Rodell, 2015; Jia et al., 2020). This is important in developing contexts like Latin America where the lack of information, representativeness, or discrimination prevents many people from accessing basic rights such as education, health, or justice, regardless of income disparities (Oxhorn, 2003; Soler‐Martínez et al., 2023). Thus, in this study we look beyond perceptions of fair income distribution and incorporate perceptions about access to fundamental rights.

## Antecedents: Social status and the moderating role of neoliberalism

Literature suggests that fairness perceptions, principally on the distributive dimension, have antecedents at both individual and societal levels. At an individual level, social status has been remarked upon as a relevant antecedent. Social status refers to the relative rank that individuals or groups have within the framework of a social system of stratification based on prestige, esteem, and respect (Cheng et al., 2014; Weiss & Fershtman, 1998). Extensive evidence from social psychology suggests that fairness perceptions vary across social status groups (Caricati & Lorenzi‐Cioldi, 2012; Castillo et al., 2022). Self‐interest theory provides a robust framework to understand these differences, suggesting that individuals fundamentally act to advance their own interests and select options that provide them with the greatest personal benefit (Miller, 1999). Thus, higher‐status individuals tend to develop more positive evaluations of justice since, in this way, they legitimize the inequalities and privileges that benefit them (Allen & Hung Ng, 2000). In this way, higher‐status individuals achieve mental consistency between their privileged position and their perception of the distribution of social resources (Rodriguez‐Bailon et al., 2017). Empirical evidence supports these theoretical expectations, as individuals from higher social classes tend to perceive greater fairness in income distribution and hold stronger meritocracy beliefs (McCoy & Major, 2007).

Other authors have suggested that low‐status people can sustain positive justice evaluations to the extent that it allows them to justify the status quo and reduce the uncertainty of change—the strong hypothesis of System Justification Theory (Jost, 2019). However, most of the comparative evidence using representative samples of countries indicates that high‐status individuals tend to develop more positive evaluations of justice (Caricati & Lorenzi‐Cioldi, 2012; Hvidberg et al., 2023; Jia et al., 2020; Rodriguez‐Bailon et al., 2017) and that the latter proposal would be observed under certain conditions (Kesberg et al., 2024). In addition, it is important to note that most of this evidence comes from developed countries. Thus, we hypothesize that higher‐status individuals will have greater perceptions of social justice in Latin America, a region where there is currently a research gap.

At a societal level, income inequality, usually measured through the Gini Index, has been positively associated with unfairness perceptions (Caricati, 2017; Reyes & Gasparini, 2022; Soler‐Martínez et al., 2023). However, the role of political and economic contexts in shaping justice perceptions remains underexplored. Neoliberalism is the world's hegemonic paradigm of economic development that encompasses both economic and ideological elements (Bettache & Chiu, 2019; England & Ward, 2016). On the one hand, neoliberalism comprises a set of economic policies (e.g. deregulation, privatization, and liberalization) and institutional arrangements to promote deregulated capital accumulation (leading to high levels of wealth and power concentration strengthening monopolies and oligopolies, that is, markets that are less competitive) and limit state involvement in the economy and social life to pursue efficiency and competitiveness (Harvey, 2005; Peck, 2017). On the other hand, these policies promote certain ideologies centred on (extreme) individual autonomy, meritocracy, and the primacy of the market (Adams et al., 2019). These ideologies have profound psychological consequences, leading people to believe that they are entirely responsible for their own lives, that they have to avoid state support, and that they must find private solutions to their problems (Peters, 2016; Pyysiäinen et al., 2017). It could be expected that people in societies that have adopted this type of economic policy may have internalized these ideas of individual responsibility and meritocracy, and thus perceive that resources are distributed and provided fairly since access to them would be attributed to the effort of people (Bettache et al., 2020). In these political contexts, these beliefs proliferate in societies, and consequently, individuals will tend to accept inequalities (Bettache et al., 2020). Therefore, it could be expected that the differences between social status groups would tend to be smaller in contexts where there is a greater adoption of these neoliberal policies. In other words, when neoliberalism is dominant, lower‐status individuals may be more likely to attribute their disadvantaged position to personal factors rather than structural inequities, thus perceiving greater justice in the system despite their position. Conversely, higher‐status individuals in neoliberal contexts have additional ideological resources to justify their privileged position, reinforcing their perception of the system as fair. Therefore, in addition to observing whether status is related to justice evaluations, we hypothesize that neoliberalism has a moderating role in that relationship. Specifically, we predict that the gap in justice perceptions between high‐ and low‐status individuals will be smaller in more neoliberal contexts.

## The political outcomes of justice evaluations

This study also explores the consequences of perceived social justice on political behaviour as this is crucial for comprehending broader sociopolitical dynamics. Different theories assume that justice assessments are relevant for social cohesion (Delhey et al., 2018), and the legitimization and justification of the system (Jost, 2019; Kesberg et al., 2024). In the social cohesion literature, perceptions of fairness are related to institutional trust in the dimension of connectedness, which is an aspect of social cohesion that looks at the relationship between individuals and their social institutions (Delhey et al., 2018). From a social psychological perspective, System Justification Theory proposes that justice evaluations are related to legitimizing attitudes toward the system (Jost, 2019), in order to preserve the status quo. Also, it has been suggested that unfairness perceptions are related to political instability and the pursuit of social change through protests (Kesberg et al., 2024; Reyes & Gasparini, 2022; Soler‐Martínez et al., 2023). Thus, examining the consequences of justice perceptions is crucial because it helps to understand attitudes that legitimize the social order or, alternatively, promote actions that lead to change.

In comparative studies on justice evaluations, legitimatizing attitudes have usually been measured with political trust indicators (e.g. Brandt, 2013; Brandt et al., 2020; García‐Sánchez et al., 2024; van der Toorn et al., 2011). It is necessary to extend this evidence to other attitudes of legitimization of the system. We hypothesized that justice evaluations are related to satisfaction with life, political and economic systems, and attitudes toward politicians (Kesberg et al., 2024). In terms of seeking social change, we also consider it important to expand the literature to other measures such as support for and the intentions to participate in protests (Kesberg et al., 2024; Soler‐Martínez et al., 2023). Finally, we are interested in the relationship between justice perceptions and life satisfaction. Political science considers life satisfaction as an important variable associated with the support of democracy, which in turn can also be associated with attitudes of legitimization and the search for social change (Welzel, 2013).

## The present study in the Latin American context

Latin America offers an interesting scenario to analyse the perceptions of social justice, its antecedents, and consequences. First, Latin America is characterized by societies with high levels of economic and social disparities (ECLAC, 2023). These disparities are not only related to income distribution but also to access to fundamental rights such as health, education, or justice. Lack of information, representativeness, or discrimination prevents many people from accessing those services (Oxhorn, 2003; Soler‐Martínez et al., 2023). These social characteristics have led many researchers to look at the association between these structural inequalities and perceptions of income justice in this region, finding a positive association (e.g. García‐Sánchez et al., 2024; Reyes & Gasparini, 2022; Zmerli & Castillo, 2015). However, it is necessary to examine whether these findings remain when considering other perceptions of social justice (Soler‐Martínez et al., 2023).

Second, this region has been marked by the debate around the adoption of neoliberalism as a development policy (Ruckert et al., 2017). Free market and privatization policies were introduced in the late 1970s in Chile, during the dictatorship of Augusto Pinochet (Harvey, 2005). Subsequently, these types of policies were introduced in most Latin American countries during the 1980s and 1990s (Ruckert et al., 2017). With the arrival of the new millennium, and after suffering severe economic crises, some governments questioned and rejected some of the policy proposals associated with neoliberalism (Ruckert et al., 2017). Thus, although this region has cultural aspects in common (e.g., language, religion, etc.), it also allows us to appreciate differences in the adoption of neoliberalism as a policy approach to boost economic growth, where there are states that reject it (e.g., Venezuela), while others continue to support it (e.g., Chile).

Third, in recent years, the countries of this region have been experiencing political turbulence, which has been characterized by the eruption of strong protests, which in some cases involved the use of violence (Murillo, 2021). Scholars in Latin America have linked these protests to people's dissatisfaction with the prevailing neoliberal model (Hechler et al., 2024), as well as economic stagnation and the reduction of inequities (Çakal et al., 2024). Therefore, this study can also contribute to shedding light on the possible psychological antecedents of this recent political instability in the region.

Drawing on the literature review and Latin American context, this study aims to study the antecedents and consequences of perceptions of social justice, through indicators that not only involve perceptions of income distribution but also fair access to fundamental rights such as education, health and justice. First, on antecedents, we hypothesize that social status is positively related to perceived social justice across the region (H1). At a country level, we expect that neoliberalism moderates the relationship between justice perceptions and social status, even controlling for Gini coefficient (H2). Second, we assume that social justice perception has a positive effect on legitimizing attitudes such as life satisfaction and satisfaction with democracy and the economic system, but a negative effect on the support for and the intention to participate in protests (H3).

## METHODS

### Participants and data

The data used in this analysis are from the Latinobarometro (2023). The current research included 20,204 participants from 18 countries: Argentina, Bolivia, Brazil, Chile, Colombia, Costa Rica, Dominican Republic, Ecuador, El Salvador, Guatemala, Honduras, Mexico, Nicaragua, Panama, Paraguay, Peru, Uruguay and Venezuela. Nicaragua was excluded as factorial models for perceived social justice did not converge for this country. The average age of this final sample was 41.27 years (SD = 16.55 years), and 52% were women (Table 1).

**TABLE 1:** Descriptives of the sample by country.

Country	*N*	Age		% Female
		*Mean*	*SD*	
Argentina	1200	42.22	15.45	53%
Bolivia	1200	38.20	15.56	50%
Brazil	1204	42.42	17.01	53%
Chile	1200	44.49	17.01	54%
Colombia	1200	40.46	16.28	52%
Costa Rica	1000	41.13	16.64	52%
Rep. Dominicana	1000	39.92	16.52	50%
Ecuador	1200	39.45	15.72	51%
El Salvador	1000	41.08	17.23	55%
Guatemala	1000	38.45	15.89	53%
Honduras	1000	37.51	15.73	53%
México	1200	43.01	16.83	49%
Panamá	1000	41.47	16.18	50%
Paraguay	1200	38.52	15.51	50%
Perú	1200	39.65	15.81	50%
Uruguay	1200	45.75	17.89	54%
Venezuela	1200	46.48	16.54	57%
Total	19,204	41.27	16.55	52%

*Note*: In the case of the sex of participants, we reported the percentage of females in the sample, and the rest of it was men.

Abbreviations: M, mean; SD, standard deviation.

### Instruments

Table 2 lists all the items of the Latinobarómetro survey and societal indicators used to address the research objectives.

**TABLE 2:** List of variables of the study.

Variable	Question	Response options
*Perceived social justice*		
Fairness perception on health	“How fair is the access to health?”	1 = “very unfair” to 4 = “Very fair”
Fairness perception on education	“How fair is the access to education?”	
Fairness perception on justice	“How fair is the access to justice?”	
Fairness perception on income distribution	“How fair is the distribution of income in (country)?”	
*Social status*		
Socioeconomic status	“…imagine a scale of 10 steps, in which on “1” are the poorest people and on “10” are the richest people, where would you place yourself?”	1 = “poorest” to 10 = “richest”
Social class	“…people sometimes describe themselves as belonging to a social class. You would describe yourself like belonging to the class…”	1 = “lower class” to 5 = “upper class”
Subjective income	“…does the salary you receive, and your total family income allow you to satisfactorily cover your needs? To reply to this question there was a Likert scale of 4 points…”	1 = “it's not enough, we have major problems” to 4 = “It's enough, we can save”
*Political behaviour outcomes*		
Satisfaction with life	“In general terms, would you say that you are satisfied with your life? Would you say that you are…?”	1 = “not at all satisficed” to 4 = “very satisfied”
Satisfaction with democracy	“In general, would you say that you are very satisfied, quite satisfied, not very satisfied or not at all satisfied with the functioning of democracy in (country)?”	1 = “not at all satisficed” to 4 = “very satisfied”
Satisfaction with economic system	“In general, would you say that you are very satisfied, quite satisfied, not very satisfied or not at all satisfied with the functioning of the economic situation in (country)?”	1 = “not at all satisficed” to 4 = “very satisfied”
Perception of progress of the country	“Would you say that this country…?”	1 = “Is in decline” to 3 = “Is progressing”
Evaluation of politicians	Generally speaking, would you say that (country) is governed for a few powerful groups in their own interest? Or is it governed for the good of all?	1 = “Powerful groups in their own interest” and 2 = “For the good of all”
Protest support	“…Tell me if you strongly agree (1), agree (2), disagree (3), or strongly disagree (4) with the protests”	1 = “Strongly disagree” to 4 = “Strongly agree”
Willing to participate in protest	“On a scale of 1–10, where 1 means ‘not at all willing’ and 10 ‘very willing’, how willing would you be to go out and march for higher wages and better conditions of work?”	1 = “Strongly disagree” to 4 = “Strongly agree”
*Individual control variables*		
Sex	Sex of participants	1 = “men” and 2 = “women”
Education	Level of education	1 = “illiterate” to 7 = “Complete university education”
Ideology	Left–right self‐position	0 = “left” to 10 = “right”
*Societal variables*		
Neoliberalism	Economic Freedom Index	0–100
Income inequality	Gini Index	0–1
Social and economic development	Human Development Index	0–1

#### Perceived social justice

We measured the perception of social justice using 4 items (Table 2) on the fairness of access to health, education, justice, and fairness of income distribution. These four items were answered on a 4‐point Likert scale. This scale was reversed to facilitate the statistical analyses and interpretation of results. These items were analysed through exploratory, confirmatory and multigroup factorial methods, and reliability analyses to examine psychometric properties to obtain an indicator of perceived social justice.

#### Social status

This variable was measured through three different items: socioeconomic status, social class, and subjective income. The first item inquires about the economic position in which the participant is located concerning the poverty and wealth groups. The second item asks about the social class with which the participant identifies. Finally, subjective income asks whether the income obtained by the participant is sufficient. The effect of each variable was measured separately. For the last two items, the scale of response was reversed.

#### Political behavioural outcomes

To measure the consequences of perceived social justice on support of the system or the seeking of social change, we selected 7 items from the Latinobarometro (2023). To observe the effect of perceptions of justice on support and satisfaction with the system, items related to satisfaction with life, satisfaction with the functioning of democracy and the economic system, the perception of progress in the country, and whether politicians acted out of self‐interest or the common interest were selected. To assess the effect of perceptions of justice on the pursuit of social change, we selected questions about support for and the willingness to participate in protests. Scales were reversed.

#### Individual control variables

Control variables were age (range: 16–100), sex (men or women), educational attainment, ideology, and religiosity. We controlled for demographic characteristics known to influence justice perceptions. Research indicates that age, sex, and educational attainment shape how individuals evaluate fairness through differential life experiences and structural positions (Davidson et al., 2011). Political ideology was included as it consistently predicts attitudes toward system legitimacy, with conservatives generally perceiving greater fairness (Jost et al., 2009). Religiosity was controlled as studies suggest religious beliefs can influence interpretations of social inequalities and attitudes toward distributive justice (García‐Sánchez et al., 2025). These controls strengthen the validity of our findings regarding status‐based differences in justice perceptions.

#### Neoliberalism

To measure neoliberalism, we used the Economic Freedom Index (The Heritage Foundation, 2023). This indicator assesses policies that ensure that individuals can exercise economic freedoms: rule of law, size of the state, limited regulation, and market openness. This index has been used in social science research to measure the adoption of neoliberalism in states (e.g. Bettache et al., 2020; Mijs et al., 2016). The range of the index is from 0, which means a repressive state, to 100, which means full economic freedom without government interference. To facilitate the interpretation of this indicator, it was standardized.

#### Other societal variables

Previous research has indicated the effects of development and income inequalities within societies on fairness perceptions. Thus, we used both the Human Development Index (HDI) and the Gini Index as controls. The first is an indicator of social and economic development that combines life expectancy, years of schooling, and gross national income per capita. The scale ranges from 0 (lowest human development) to 1 (highest human development). Countries with scores closer to 1 have higher levels of life expectancy, education, and income per capita. We obtained this measure from the United Nations Development Programme data centre (United Nations, 2024). The second indicator measures the income distribution in a society in a range from 0, which means “no inequalities” to 1, which means that society is fully unequal. We took this data from the Economic Commission for Latin America (2023). In a similar way to the previous indicator, the Gini Index and HDI were standardized.

### Procedure and analytic techniques

The Latinobarometro (2023) data were downloaded, reviewed, and recoded as necessary. With this, statistical analyses were performed using Stata 18 software. First, we developed our measure of perceived social justice through factorial methods. We randomly split the data, and with one half of it, we ran an exploratory (Fabrigar et al., 1999) and with the other half a confirmatory (Brown, 2015) factor analysis. Then, we ran multigroup factor analyses (Nießen et al., 2020) to determine if the configuration and the meaning of the model of perceived social justice were equal across countries. We tested two levels of invariance: configural and metric invariance. The configural analyses measured if the number of factors replicated across groups. This level of invariance is achieved when the model shows good fit indicators (Hu & Bentler, 1999). Metric invariance measures if factor loadings are consistent across the groups. Considering the large number of group comparisons in this study (Rutkowski & Svetina, 2014), this kind of invariance is rejected if there are differences in the model fit indicators (Rutkowski & Svetina, 2014): ΔCFI ≤ −.020 and ΔRMSEA ≥ .015 or SRMR ≥ .030. To address possible problems in reaching invariance, we ran modification indices analyses (Sörbom, 1989). To obtain scores for each factor, we chose to use the average of the four items.

To examine the moderating role of neoliberalism on the relationship between social status and social justice perceptions, we employed a systematic multilevel modelling approach appropriate for our nested data structure (individuals within countries). First, we established baseline models with random effects, confirming significant between‐country variation in justice perceptions. We then tested a random slope model allowing the effect of social status to vary across countries. The significant variation in these slopes provided empirical justification for exploring cross‐level interactions.

Following this preliminary phase, we proceeded to mixed‐effects models incorporating country‐level predictors. We first tested the direct effect of economic freedom (our operationalization of neoliberalism) on justice perceptions. We then expanded this model by incorporating additional country‐level controls (Gini coefficient and Human Development Index) to account for potential confounding factors. In the final analytical step, we specified cross‐level interaction models to formally test whether country‐level economic freedom moderates the individual‐level association between social status and justice perceptions. This was accomplished by including an interaction term between social status and economic freedom while maintaining the appropriate control variables at both levels of analysis.

All models were estimated using maximum likelihood procedures, and model fit was assessed through likelihood ratio tests and information criteria (AIC and BIC). This sequential approach allowed us to systematically evaluate our hypothesized moderating effect while controlling for relevant covariates.

## RESULTS

### Perceptions of social justice

**FIGURE 1:**
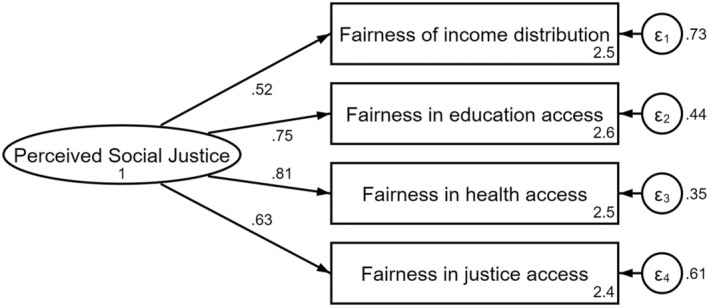
Confirmatory factor analysis of perceived social justice.

The results suggest that perceived social justice can be summarized as a latent factor for almost all Latin American countries. The exploratory factor analysis applied to one half of the sample suggests that the four items of fairness perceptions group in only one dimension (Appendix A). The confirmatory factor analysis on the other half of the sample suggests that the single‐factor solution is acceptable (χ^2^(2) = 129.30, *p* < .00; CFI = .987; RMSEA = .083, SRMR = .022, Figure 1), although it exceeds the acceptance threshold for the RMSEA (Hu & Bentler, 1999). This analysis indicates that education and health access are the most relevant variables in this perception of social justice for Latin America, which coincides with the evidence that finds that these variables are among the most important justice concerns in this region (Soler‐Martínez et al., 2023). Following Nießen et al. (2020), multigroup confirmatory factor analyses indicate that not all goodness‐of‐fit indicators present acceptable values to achieve configural invariance (χ^2^(36) = 324.92, *p* < .00; CFI = .982; RMSEA = .088, SRMR = .026). Then, we ran a modification indices analysis to identify improvements for the factor analyses (Appendix C). Results suggest that Nicaragua had the highest modification indices, so it was decided to withdraw this country from the subsequent invariance analyses. With this, the next multigroup factor analysis suggests that configural invariance was achieved for the rest of the countries (χ^2^(34) = 256.56, *p* < .00; CFI = .985; RMSEA = .079, SRMR = .025). In terms of metric invariance, we can assume acceptable fit indices (χ^2^(82) = 591.90, *p* < .00; CFI = .966; RMSEA = .077, SRMR = .057). On average, the Cronbach alpha for these items across countries is 0.72 (min. 0.62/max. 0.86). Thus, it is plausible to assume that these four items represent a general perception of system fairness which has a similar meaning across Latin America.

**FIGURE 2:**
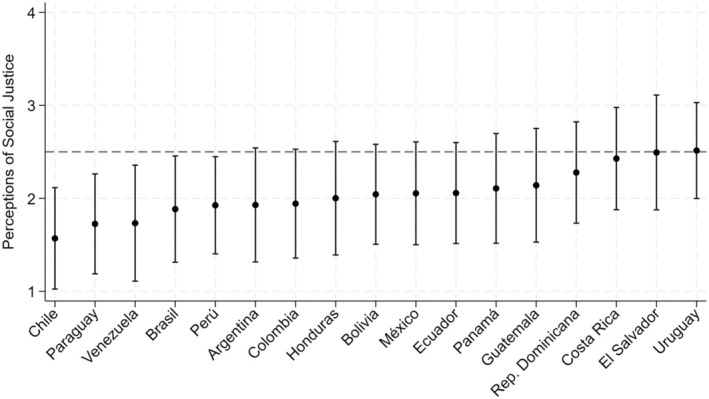
Means and error bars of Perceived Social Justice by country.

Descriptive analyses suggest that in most Latin American countries there is a general perception of social injustice (Figure 2), results that are in line with previous evidence in this region on distributive fairness (García‐Sánchez et al., 2024; Soler‐Martínez et al., 2023). These perceptions are mainly driven by negative evaluations on the access to justice and income distribution (Appendix B). Perceptions of justice on access to education and health are more favourable in most countries in the region, which reinforces the idea that the study of social justice must consider not only aspects related to the distribution of income resources (Soler‐Martínez et al., 2023).

### The role of social status on perceived social justice

The mixed models suggest that perceived social justice is affected by social status (Table 3). First, we ran a null model to look at the differences in perceptions of justice between countries. Results clearly suggest that there are significant differences between them. On average, socioeconomic status, social class and subjective income have positive relationships with perceived social justice. Random‐effects models with random intercepts and slopes, which allow us to see the variation in the relationships between perceptions of justice and social status among different groups, suggest that the level of these relationships varies among Latin American countries, which is in line with our premise that these relations are moderated by neoliberalism. In general, it can be pointed out that social status influences fairness perceptions in Latin America, including other aspects of inequality like income distribution, a finding that expands the current evidence (Caricati, 2017). Other interesting results indicate that right‐wing political positions, being men, and being older tend to be associated with a greater perception of social justice. It can also be observed that education has a negative relationship with these perceptions.

**TABLE 3:** Mixed models on the role of social status on perceived social justice.

	Baseline	Perceived social justice					
		Random effects	Random slopes	Random effects	Random slopes	Random effects	Random slopes
Socioeconomic status		0.017***	0.019***				
		(0.002)	(0.005)				
Social class				0.058***	0.059***		
				(0.005)	(0.008)		
Subjective salary						0.058***	0.063***
						(0.005)	(0.011)
Ideology		0.009***	0.010***	0.009***	0.009***	0.010***	0.011***
		(0.002)	(0.002)	(0.002)	(0.002)	(0.002)	(0.002)
Education		−0.030***	−0.030***	−0.029***	−0.029***	−0.032***	−0.032***
		(0.003)	(0.003)	(0.003)	(0.003)	(0.003)	(0.003)
Sex (2 = women)		−0.046***	−0.046***	−0.043***	−0.043***	−0.038***	−0.039***
		(0.009)	(0.009)	(0.009)	(0.009)	(0.009)	(0.009)
Age		−0.003***	−0.003***	−0.003***	−0.003***	−0.003***	−0.003***
		(0.000)	(0.000)	(0.000)	(0.000)	(0.000)	(0.000)
Constant	2.048***	2.281***	2.269***	2.209***	2.206***	2.201***	2.188***
	(0.062)	(0.064)	(0.067)	(0.063)	(0.067)	(0.064)	(0.070)
var(intercept)	0.066***	0.057***	0.065***	0.056***	0.063***	0.056***	0.07***
	(.0227)	(.019)	(023)	(.0192)	(.023)	(.019)	(.025)
var(residual)	0.324***	0.315***	0.314***	0.312***	0.311***	0.313***	0.312***
	(.003)	(.004)	(.004)	(.004)	(.004)	(.004)	(.004)
var(slope)			0.000***		0.001***		0.002***
			(0.000)		0.000		(0.001)
*N*	19,088	15,430	15,430	15,053	15,053	15,213	15,213
ICC	0.169	0.153	0.172	0.151	0.169	0.152	0.183
Log likelihood	−16,360.19	−13,021.57	−13,007.26	−12,631.04	−12,626.58	−12,796.59	−12,781.48
AIC	32,726.4	26,059.1	26,032.5	25,278.1	25,271.2	25,609.2	25,581.0
BIC	32,750.0	26,120.3	26,101.3	25,339.0	25,339.7	25,670.2	25,649.6

*Note*: Standard errors in parentheses.

Abbreviations: AIC, Akaike Information Criterion; BIC, Bayesian Information Criterion; ICC, interclass correlation coefficient; *N*, number of participants.

***p < .001.

**TABLE 4:** Mixed‐effects models on neoliberalism and societal variables on perceived social justice.

	Perceived social justice					
	Model 1	Model 2	Model 3	Model 4	Model 5	Model 6
Socioeconomic status	0.017***	0.017***				
	(0.002)	(0.002)				
Social class			0.058***	0.058***		
			(0.005)	(0.005)		
Subjective salary					0.057***	0.057***
					(0.005)	(0.005)
Ideology	0.009***	0.009***	0.009***	0.009***	0.010***	0.010***
	(0.002)	(0.002)	(0.002)	(0.002)	(0.002)	(0.002)
Education	−0.030***	−0.030***	−0.029***	−0.029***	−0.032***	−0.032***
	(0.003)	(0.003)	(0.003)	(0.003)	(0.003)	(0.003)
Sex (2 = women)	−0.046***	−0.046***	−0.043***	−0.043***	−0.038***	−0.038***
	(0.009)	(0.009)	(0.009)	(0.009)	(0.009)	(0.009)
Age	−0.003***	−0.003***	−0.003***	−0.003***	−0.003***	−0.003***
	(0.000)	(0.000)	(0.000)	(0.000)	(0.000)	(0.000)
Economic Freedom Index	0.050	0.143*	0.048	0.140*	0.043	0.135*
	(0.057)	(0.064)	(0.056)	(0.063)	(0.057)	(0.064)
Gini Index		−0.145*		−0.145*		−0.145*
		(0.059)		(0.058)		(0.059)
Human Development Index		−0.042		−0.040		−0.039
		(0.054)		(0.053)		(0.054)
Constant	2.280***	2.273***	2.207***	2.201***	2.200***	2.193***
	(0.063)	(0.055)	(0.062)	(0.055)	(0.063)	(0.056)
var(intercept)	0.055***	0.040***	0.053***	0.039***	0.054***	0.040***
	(0.019)	(0.014)	(0.018)	(0.013)	(0.019)	(0.014)
var(residual)	0.315***	0.315***	0.312***	0.312***	0.313***	0.313***
	(0.004)	(0.004)	(0.004)	(0.004)	(0.004)	(0.004)
*N*	15,430	15,430	15,053	15,053	15,213	15,213
ICC	0.148	0.113	0.146	0.111	0.148	0.113
Log likelihood	−13,021.194	−13,018.572	−12,630.678	−12,628.010	−12,796.302	−12,793.672
AIC	26,060.39	26,059.14	25,279.36	25,278.02	25,610.60	25,609.34
BIC	26,129.18	26,143.23	25,347.93	25,361.83	25,679.27	25,693.27

*Note*: Standard errors in parentheses.

Abbreviations: AIC, Akaike Information Criterion; BIC, Bayesian Information Criterion; ICC, interclass correlation coefficient; N, number of participants.

*p < .05; ***p < .001.

### Neoliberalism and societal variables on perceived social justice

First, we conducted mixed models without interactions to control for the possible relationship between indicators of neoliberalism and perceptions of social justice (Table 4). Only when the three contextual variables are incorporated do both the income inequalities (Gini) and the economic freedoms (neoliberalism) indicators have a significant relationship with perceptions of justice. These results could be a first indication of the relationship between neoliberalism and perceptions of justice when other contextual factors are controlled. Likewise, these results also align with previous evidence suggesting that neoliberalism affects perceptions of inequality (Bettache et al., 2020). However, this is still very limited evidence and the greatest interest of the study is on the possible moderating role of neoliberalism. The level of human development does not seem to have a significant effect, and the Gini coefficient has a significant and negative relationship with perceptions of social justice. Results are in line with previous evidence regarding Gini and its effects on perceptions of inequities in people's income distribution (García‐Sánchez et al., 2024; Reyes & Gasparini, 2022; Zmerli & Castillo, 2015). Finally, it is important to note that the inclusion of neoliberalism and societal variables in the model did not affect the relationships between social status variables or other control variables.

**FIGURE 3:**
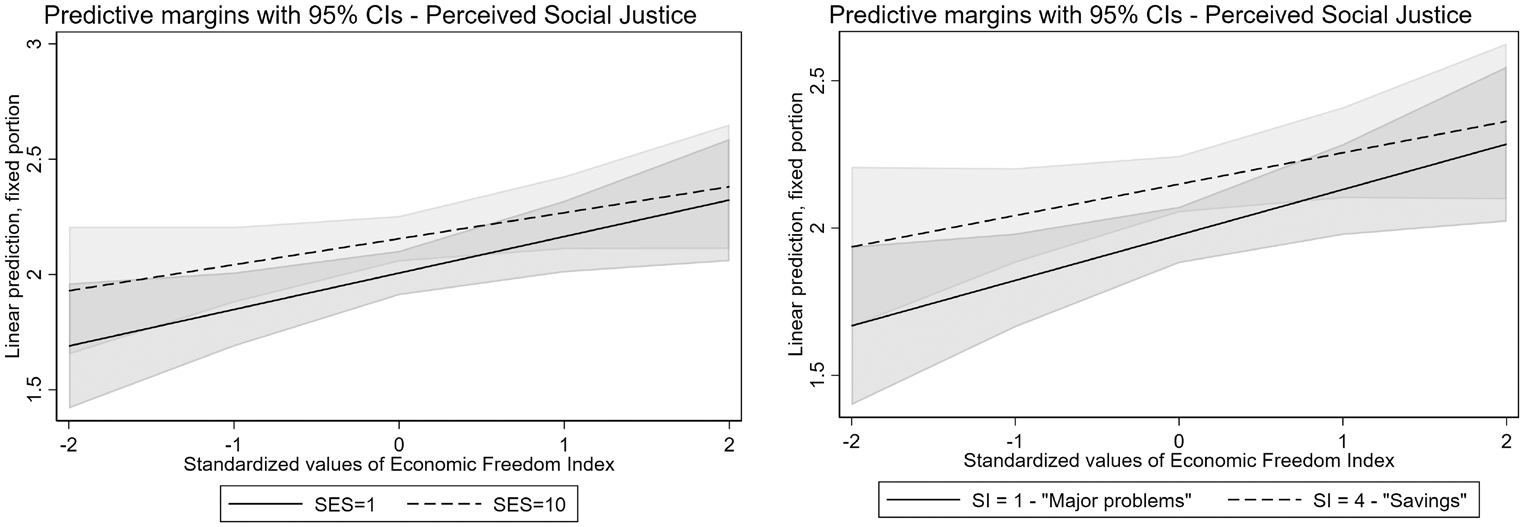
Predictive margins between social status and economic freedom index on perceived social justice. SES, socioeconomic status (1 = lowest, 10 = highest); SI, subjective income. The shaded areas represent the confidence intervals at 95% of the predictive margins.

Then, we investigated the interaction between the neoliberal context and social status. The results indicate that there are interactions between neoliberalism on two of our three indicators of social status: socioeconomic status and subjective income (Table 5). Based on these results, our hypothesis that the economic freedom context moderates the relationship between social status and perceptions of social justice is mostly supported. The negative sign of these interactions suggests that the positive effect of social status on perceptions of social justice decreases as a country's economic freedom increases, indicating a moderating effect of the institutional context as we expected (Figure 3). Furthermore, the reduction in Intraclass Correlation Coefficient when contextual variables are included demonstrates that some of the between‐country variation in social justice perceptions can be explained by these contextual factors and their interactions with individual characteristics.

**TABLE 5:** Mixed‐effects models on the interaction between neoliberalism and social status indicators on Perceived Social Justice.

	Perceived social justice					
	Model 1	Model 2	Model 3	Model 4	Model 5	Model 6
Socioeconomic status (SES)	0.017***	0.017***				
	(0.002)	(0.002)				
Interaction SES × EFI	−0.005*	−0.005*				
	(0.002)	(0.002)				
Social class			0.058***	0.058***		
			(0.005)	(0.005)		
Interaction Social class × EFI			−0.007	−0.007		
			(0.005)	(0.005)		
Subjective salary					0.058***	0.058***
					(0.005)	(0.005)
Interaction sub. salary × EFI					−0.016**	−0.016**
					(0.006)	(0.006)
Ideology	0.010***	0.010***	0.009***	0.009***	0.010***	0.010***
	(0.002)	(0.002)	(0.002)	(0.002)	(0.002)	(0.002)
Education	−0.030***	−0.030***	−0.029***	−0.029***	−0.032***	−0.032***
	(0.003)	(0.003)	(0.003)	(0.003)	(0.003)	(0.003)
Sex (2 = women)	−0.046***	−0.046***	−0.043***	−0.043***	−0.039***	−0.039***
	(0.009)	(0.009)	(0.009)	(0.009)	(0.009)	(0.009)
Age	−0.003***	−0.003***	−0.003***	−0.003***	−0.003***	−0.003***
	(0.000)	(0.000)	(0.000)	(0.000)	(0.000)	(0.000)
Economic Freedom Index (EFI)	0.070	0.163*	0.064	0.157*	0.078	0.170**
	(0.058)	(0.064)	(0.057)	(0.063)	(0.058)	(0.064)
Gini Index		−0.146*		−0.145*		−0.146*
		(0.059)		(0.058)		(0.058)
Human Development Index		−0.042		−0.039		−0.038
		(0.054)		(0.053)		(0.054)
Constant	2.280***	2.273***	2.208***	2.201***	2.201***	2.194***
	(0.062)	(0.055)	(0.062)	(0.055)	(0.063)	(0.056)
var(intercept)	0.054***	0.040***	0.053***	0.039***	0.054***	0.039***
	(0.019)	(0.014)	(0.018)	(0.013)	(0.019)	(0.014)
var(residual)	0.315***	0.315***	0.312***	0.312***	0.313***	0.313***
	(0.004)	(0.004)	(0.004)	(0.004)	(0.004)	(0.004)
*N*	15,430	15,430	15,053	15,053	15,213	15,213
ICC	0.147	0.112	0.146	0.111	0.147	0.111
Log likelihood	−13,018.983	−13,016.342	−12,629.515	−12,626.837	−12,792.225	−12,789.536
AIC	26,057.97	26,056.68	25,279.03	25,277.67	25,604.45	25,603.07
BIC	26,134.41	26,148.41	25,355.22	25,369.11	25,680.75	25,694.63

*Note*: Standard errors in parentheses.

Abbreviations: AIC, Akaike Information Criterion; BIC, Bayesian Information Criterion; ICC, interclass correlation coefficient; *N*, number of participants.

*p < .05; **p < .01; ***p < .001.

In summary, the results suggest that both social status and neoliberal contexts moderate perceptions of social justice, indicating that in neoliberal contexts differences in perceptions of justice tend to narrow between social status groups.

### The political behavioural outcomes of perceived social justice

Finally, we included the variable of perceived social justice as an independent variable to measure its effect on several political behavioural outcomes (Table 6). Mixed models suggest that those who perceive higher levels of social justice in Latin America tend to, on average, be more satisfied with democracy, with the economic system, and with life, in addition to having a better perception of the progress of the country and the country's politicians. On the other hand, perceived social justice has a negative effect on support for protests and the intention to participate in protests. In other words, perceived social justice is one of the most relevant psychological aspects associated with the legitimacy and support of political systems, the maintenance of the status quo, and with reducing the tendency to support actions to seek social change in Latin America.

**TABLE 6:** Mixed‐effects models of the influence of perceived social justice on political outcomes.

	Satisfaction with democracy	Satisfaction with economic system	Life satisfaction	Perception of country progress	Evaluation of politicians	Protest support	Willing to participate in protest
Perceived social justice	0.397***	0.375***	0.154***	0.240***	0.162***	−0.068***	−0.094***
	(0.012)	(0.011)	(0.012)	(0.009)	(0.006)	(0.011)	(0.012)
Socioeconomic status	0.037***	0.040***	0.053***	0.022***	−0.001	−0.007*	0.010**
	(0.004)	(0.003)	(0.004)	(0.003)	(0.002)	(0.003)	(0.004)
Ideology	0.010***	0.011***	−0.002	0.006**	0.011***	−0.011***	0.003
	(0.002)	(0.002)	(0.002)	(0.002)	(0.001)	(0.002)	(0.002)
Education	−0.012**	−0.006	0.039***	−0.004	−0.029***	0.043***	0.004
	(0.004)	(0.004)	(0.004)	(0.003)	(0.002)	(0.004)	(0.005)
Sex (2 = women)	−0.031*	−0.062***	−0.004	−0.025*	0.032***	−0.087***	−0.102***
	(0.014)	(0.012)	(0.013)	(0.010)	(0.006)	(0.013)	(0.014)
Age	0.003***	−0.000	−0.002***	0.000	0.001*	−0.005***	−0.007***
	(0.000)	(0.000)	(0.000)	(0.000)	(0.000)	(0.000)	(0.000)
Gini Index	−0.186***	−0.069	−0.035	−0.077	−0.065**	−0.004	−0.030**
	(0.046)	(0.045)	(0.046)	(0.050)	(0.022)	(0.038)	(0.010)
Human Development Index	−0.009	−0.079^+^	−0.126**	−0.084+	−0.027	0.100**	−0.005
	(0.043)	(0.041)	(0.043)	(0.046)	(0.020)	(0.035)	(0.010)
Economic Freedom Index	0.188***	0.167***	0.142**	0.137*	0.038	0.040	0.016
	(0.050)	(0.049)	(0.050)	(0.054)	(0.023)	(0.041)	(0.011)
Constant	1.029***	0.947***	2.504***	1.314***	0.903***	2.945***	0.592***
	(0.061)	(0.056)	(0.060)	(0.056)	(0.029)	(0.055)	(0.050)
var(intercept)	0.024***	0.023***	0.024***	0.029***	0.005***	0.016***	0.000***
	(0.009)	(0.008)	(0.009)	(0.010)	(0.002)	(0.006)	(0.001)
var(residual)	0.699***	0.538***	0.668***	0.410***	0.148***	0.623***	0.783***
	(0.008)	(0.006)	(0.008)	(0.005)	(0.002)	(0.007)	(0.009)
*N*	15,015	15,129	15,322	15,066	14,859	15,030	15,430
ICC	0.033	0.041	0.035	0.066	0.034	0.025	0.000
Log likelihood	−18,647.927	−16,803.902	−18,675.749	−14,699.853	−6930.871	−17,799.405	−20,013.985
AIC	37,319.85	33,631.80	37,375.50	29,423.71	13,885.74	35,622.81	40,051.97
BIC	37,411.26	33,723.30	37,467.14	29,515.15	13,977.02	35,714.22	40,143.70

*Note*: Standard errors in parentheses.

Abbreviations: AIC, Akaike Information Criterion; BIC, Bayesian Information Criterion; ICC, Interclass correlation coefficient; N, number of participants.

*p < .05; **p < .01; ***p < .001; ^+^ *p* < .10.

## CONCLUSIONS AND DISCUSSION

In this study, we measured perceptions of social justice, including not only subjective evaluations of income distribution, but also the perceptions of access to other fundamental rights. Results support the idea that perceptions of justice in access to justice, education and health are relevant elements of social justice in Latin America (Soler‐Martínez et al., 2023). In a context with high levels of social and economic disparities, this research highlights the need to continue studying perceptions of justice beyond income disparities and consider access to fundamental rights.

This research also contributes to understanding the relevance of social status in shaping perceptions of justice. Following the theory of self‐interest (Miller, 1999), we conclude that people develop beliefs and attitudes about social justice according to the circumstances that suit them best (Allen & Hung Ng, 2000). In our research, we found that people with higher status tend to have a vision of greater justice in society, which allows them to maintain beliefs that justify the status quo and its privileges. These findings challenge the discussion of the strong hypothesis of System Justification Theory, which suggests that low‐status individuals tend to appreciate social systems as fairer compared to high‐status ones (Jost et al., 2003). Some scholars have employed measures of fairness perceptions of income distribution as a measure of System Justification (Caricati, 2017; Caricati & Lorenzi‐Cioldi, 2012), as perceptions of fairness of political, economic, and social systems are part of this concept (Árnadóttir et al., 2024). From this perspective, accumulating evidence suggests that, in representative samples around the world, low‐status people do not tend to consider their societies as fair, at least in terms of income distribution (Caricati, 2017; Caricati & Lorenzi‐Cioldi, 2012). Future studies in Latin America should delve into this relationship by examining individual countries since our results suggest that this relationship is not homogeneous across these societies.

A third key finding is the moderating relationship between neoliberalism and perceptions of social justice. Previous evidence in the region has delved into the relationships between perceptions of income distribution and the Gini index (García‐Sánchez et al., 2024; Reyes & Gasparini, 2022). In this study, we argue that neoliberalism is associated with social justice beliefs as this economic development paradigm assumes that individuals are solely responsible for their life and therefore that their social position is a manifestation of their efforts – meritocracy beliefs (England & Ward, 2016), which can affect their perceptions of fairness (Bettache et al., 2020). In this sense, this study contributes to recent advances on the effects of neoliberalism on the social psychology of societies. Even though Latin America is a region that is quite influenced by neoliberal policies, their effects have not been properly examined. The results indicate that in societies with higher levels of neoliberalism, people from different social status groups tend to have more similar perceptions of justice, compared to countries with less neoliberal policy adoption. This finding has relevant implications for development politics as the system justifying ideas that neoliberalism promotes facilitate the maintenance of this system; in other words, they promote the status quo. In addition, this result also has important implications for System Justification Theory as the neoliberal context could be a variable that makes the strong hypothesis of this theory more plausible. Future studies should continue to study these relationships and analyse whether other variables may be mediating the effects between neoliberalism and perceptions of justice (e.g. meritocracy beliefs).

In terms of political outcomes of perceived social justice, this research expands the literature in Latin America, which has focused on trust (e.g. García‐Sánchez et al., 2024; Zmerli & Castillo, 2015). We find that fairness perceptions also affect attitudes that legitimize political systems and support the status quo, such as satisfaction with democracy, economic system, life satisfaction, perception of country progress, and positive evaluation of politicians. On the other hand, our results indicate that perceived social justice is also related to behaviours that challenge the political systems, such as supporting protests and the intention to participate in them. Therefore, we can conclude that perceptions of social justice are critical to both the reproduction and the transformation of political systems.

In a context of political turmoil (as seen in recent years in Latin America), this research provides valuable insights. Between 2019 and 2021, a series of social protests erupted in different countries in the region, where the main demands targeted the economic adjustments that reversed some social improvements promoted by policies during the years of economic growth that preceded the crisis (Murillo, 2021). The economic stagnation was also exacerbated by the arrival of the Covid‐19 pandemic, where the populations of lower social status were affected the most (Murillo, 2021). As we can see in this research, these social groups have lower levels of perception of justice in their respective societies, which could explain the fact that these protests had stronger social support and participation from these groups than elites (Murillo, 2021). Thus, this research also contributes to approaching the Latin American political crisis from a psychological perspective.

In summary, this work contributes to understanding how perceptions of social justice can contribute to the maintenance of the status quo and how these perceptions are affected by people's social status and neoliberal contexts.

This research has important limitations, which future studies should address. First, although it is a step forward to evaluate other aspects of perceptions of justice such as access to basic services, this research is limited to the questions posed by the Latinobarómetro survey. Future studies could develop more specific tools to measure the different aspects that encompass perceptions of social justice. It would also be relevant to study more deeply how these relationships of perceived social justice and social status may be mediated by meritocratic beliefs. Unfortunately, this was not possible in this research, but it would be very useful in future studies. These findings also need to be replicated in other developing contexts, one of the current weaknesses of psychology, to confirm the robustness of these findings.

### AUTHOR CONTRIBUTIONS

**Dante Solano‐Silva:** Conceptualization; investigation; methodology; writing – review and editing; writing – original draft; supervision; software. **César Guadalupe:** Investigation; supervision; writing – review and editing; methodology. **Eileen Sam‐Castañeda:** Writing – review and editing; investigation; project administration; data curation.

## CONFLICT OF INTEREST STATEMENT

The authors declare that there are no conflicts of interest regarding the publication of this article.

## Supporting information


Appendix S1.

Appendix S2.

## Data Availability

The data that support the findings of this study are available in Latinobarometro at https://www.latinobarometro.org/latContents.jsp. These data were derived from the following resources available in the public domain: Latinobarometro, https://www.latinobarometro.org/latContents.jsp.
