# Supplementary material for: Ni justo ni legítimo: The role of social status and neoliberal context on perceived social justice in Latin America and its political consequences
Source: Br J Soc Psychol. 2025 Apr 28;64(3):e12894. doi: 10.1111/bjso.12894 (PMC12038224; doi:10.1111/bjso.12894)
Supplement: Supplementary file 1 — Appendix S1. [file BJSO-64-0-s002.docx]

**Appendix A – Exploratory factor analysis results**

| Factor analysis/correlation Number of obs. = 9,194 | | | | | |
| --- | --- | --- | --- | --- | --- |
| Method: principal factors Retained factors = 1 | | | | | |
| Rotation: (unrotated) Number of params = 4 | | | | | |
|  |  |  |  |  |  |
| Factor | Eigenvalue | Difference | Proportion | Cumulative | |
| Factor1 | 1.770 | 1.793 | 1.230 | 1.230 |  |
| Factor2 | -0.024 | 0.091 | -0.016 | 1.213 |  |
| Factor3 | -0.114 | 0.079 | -0.079 | 1.134 |  |
| Factor4 | -0.193 | . | -0.134 | 1.000 |  |
| LR test: independent vs. saturated: chi2(6) = 1.0e+04 Prob>chi2 = 0.0000 | | | | | |
|  |  |  |  |  |  |
| Variable | Factor1 | Uniqueness |  |  |  |
| Income | 0.537 | 0.712 |  |  |  |
| Education | 0.715 | 0.489 |  |  |  |
| Health | 0.752 | 0.435 |  |  |  |
| Justice | 0.637 | 0.595 |  |  |  |

**Appendix B – Graph of means of the four items of Perceived Social Justice by country**


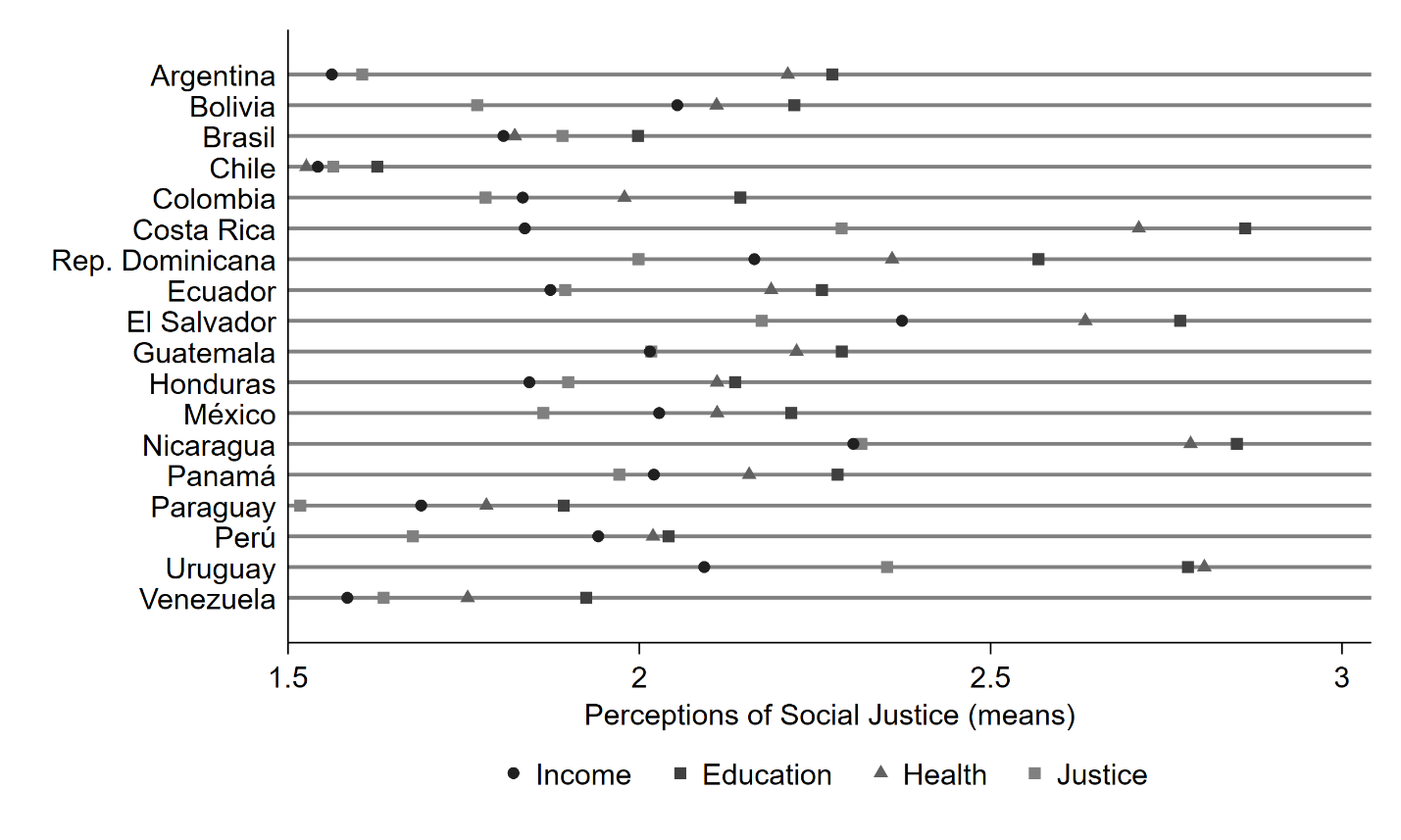


**Appendix C – Modification indices – Configural Invariance**
