# Supplementary figures and images for: Ni justo ni legítimo: The role of social status and neoliberal context on perceived social justice in Latin America and its political consequences
Source: Br J Soc Psychol. 2025 Apr 28;64(3):e12894. doi: 10.1111/bjso.12894 (PMC12038224; doi:10.1111/bjso.12894)

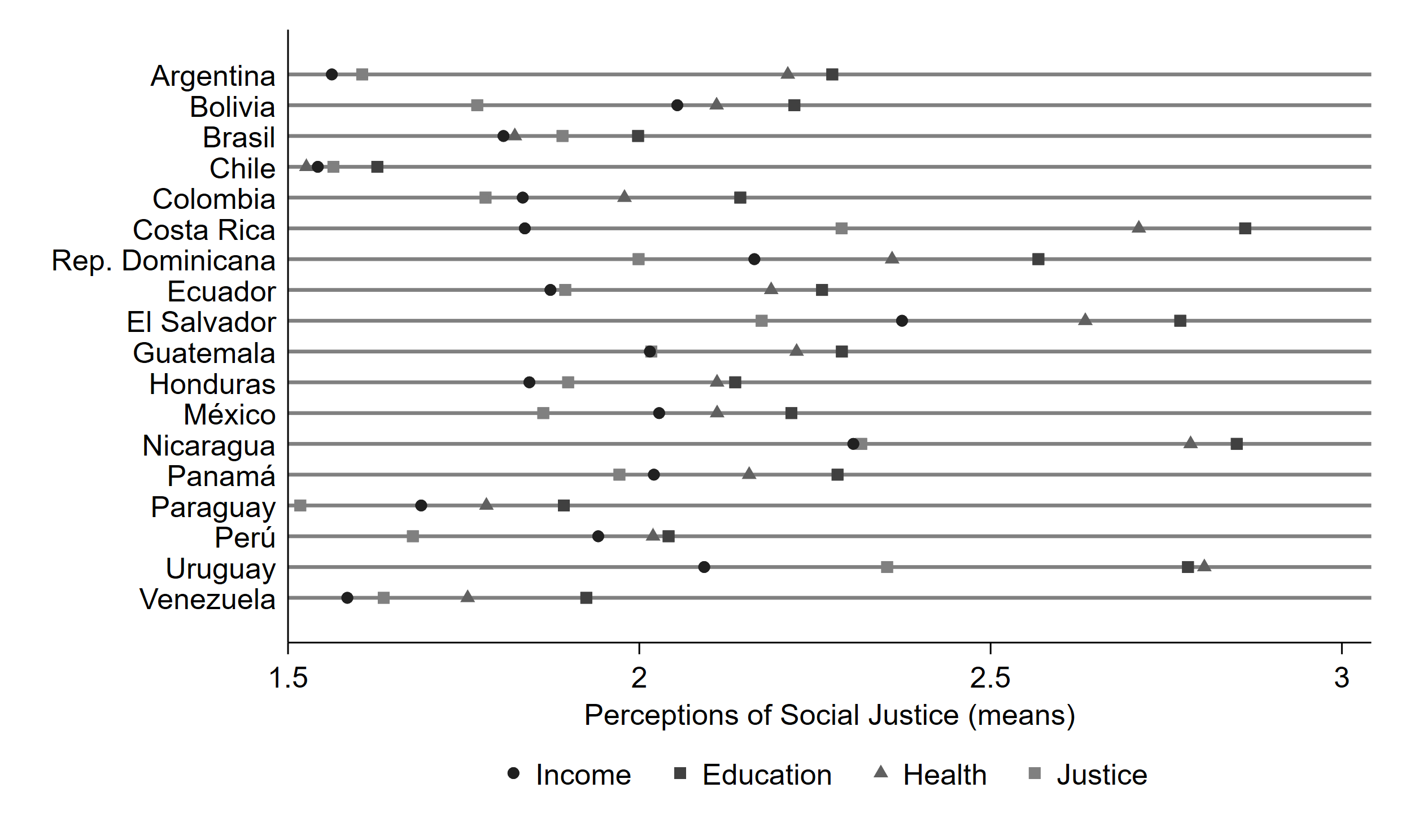

Supplement: Supplementary file 2 — Appendix S2. [file BJSO-64-0-s001.png]
